# Predictors of Endothelial Cell Loss after Phacoemulsification for the Treatment of Primary Angle Closure

**DOI:** 10.1155/2019/6368784

**Published:** 2019-07-28

**Authors:** Carlo Alberto Cutolo, Chiara Bonzano, Carlo Catti, Alessandro Bagnis, Riccardo Scotto, Letizia Negri, Sara Olivari, Francesca Cappelli, Carlo Enrico Traverso

**Affiliations:** Clinica Oculistica, DiNOGMI, University of Genoa and IRCCS San Martino Policlinic Hospital, Genova, Italy

## Abstract

**Purpose:**

To investigate demographic and anatomical factors associated with a reduction in endothelial cell density (ECD) after phacoemulsification (PE) for the treatment of primary angle closure (PAC).

**Methods:**

In this prospective case series, ECD was evaluated by noncontact specular microscopy and biometric parameters by both noncontact optical biometry and anterior segment optical coherence tomography, preoperatively and at 12 months after surgery. Anterior segment biomicroscopy and gonioscopy were also performed. The change in ECD and its relation to clinical characteristics and biometric parameters were evaluated by linear regression analysis.

**Results:**

44 patients with PAC were included in the study. The mean (SD) patient age was 71.6 (10.2) years; thirty-one (70.5%) of them were women. Coexistence of exfoliation syndrome (XS) was observed in 4 cases (9.1%). The mean (SD) ECD (cells/mm^2^) changed from 2275 (463) preoperatively to 1964 (613) postoperatively with a mean reduction of −310 (95% CI −445 to −176; *p* < 0.001). In the multivariate regression model, after correction for age and lens status, XS was the only parameter associated with ECD percentage change (*B* = −36.00; *p*=0.001).

**Conclusion:**

PE in angle closure causes a significant ECD reduction. In our population of PAC patients, XS is significantly associated with ECD change. In this group of patients, a careful preoperative endothelial evaluation should be performed.

## 1. Introduction

The worldwide prevalence of primary angle-closure glaucoma (PACG) is estimated to increase from 20 to 32 million in 2040 [1]. Compared to primary open-angle glaucoma, PACG is more likely to result in irreversible blindness if not adequately treated [2].

During the last 15 years, lens extraction, both cataract and clear lenses, has gained popularity, and a consistent amount of studies have shown the beneficial role of phacoemulsification (PE) and intraocular lens (IOL) implantation for the treatment of PACG [3–6]. Compared to iridotomy that only resolves the relative pupillary block, PE with the replacement of the natural lens with a significantly thinner artificial IOL effectively debulks the anterior segment, favoring the opening of the angle [7]. A widely accepted approach is that if IOP remains poorly controlled after iridotomy and IOP-lowering drugs, lens extraction can be considered [8, 9].

Recently, the Eagle trial provided solid evidence that PE in a selected group of patients affected by PACG or PAC with elevated IOP is associated with better quality of life, lower IOP, and less need for filtering procedures compared to iridotomy [10]. However, the Eagle study was not designed to address endothelial damage and did not include patients with lens opacity [11].

In a previous study, we showed that while PE for the treatment of PAC and PACG has a beneficial effect on IOP, it is also associated with a substantial reduction in endothelial cell density (ECD) [12]. A similar ECD decrease after PE was also reported in Asian patients affected by PACG [13].

When considering PE for the treatment of PAC and PACG, ECD evaluation may be clinically relevant because eyes with angle closure often have a reduced ECD [14]. Low ECD can prolong visual recovery after PE and can eventually trigger permanent corneal decompensation [11, 15–17].

Our study is aimed at investigating the demographic and ocular characteristics that are associated with the loss of corneal endothelial cells after PE for the treatment of PAC and PACG.

## 2. Subjects and Methods

This study is part of a prospective interventional study conducted at Clinica Oculistica, Ospedale Policlinico San Martino, University of Genoa, Italy [12]. The study enrolled patients submitted to PE and IOL implantation for the treatment of PAC and PACG. The study was approved by a qualified body and followed the tenets of the Declaration of Helsinki. PAC was diagnosed by the presence of two or more quadrants of iridotrabecular contact combined with either elevated IOP or peripheral anterior synechiae, or both. The term “glaucoma” was added if glaucomatous optic neuropathy was present. For the analysis, all patients with a reliable endothelial specular microscopy before and at one year after PE were included in the study.

Inclusion criteria were the presence of PAC and the willingness to undergo the supplementary examinations. All patients underwent a full ophthalmic evaluation that included best-corrected visual acuity, slit lamp examination, fundoscopy, tonometry, dynamic gonioscopy, and visual field exam. Patients with phacodonesis or secondary angle-closure glaucoma were excluded. The presence of exfoliation material on the anterior capsule of the lens was recorded but was not considered an exclusion criterion.

Endothelial cells were evaluated by noncontact specular microscopy (Konan Specular Microscope, Konan Medical Inc., Hyogo, Japan). Anterior segment OCT (AS-OCT) was used for measurement of the central corneal thickness (CCT) and for the evaluation of the anterior chamber angle (RTVue, Optovue, Inc., Fremont, CA). Schwalbe's line-angle-opening distance (SL-AOD) and Schwalbe's line-trabecular iris space area (SL-TISA) were used as AS-OCT morphometric angle parameters [18, 19]. Optical biometry by Lenstar LS 900 (Haag-Streit AG) was used for IOL calculation and the recording of axial length, aqueous depth (AD), and lens thickness. AD is intended as the distance from the central corneal endothelium to the anterior capsule of the lens or anterior chamber depth (ACD) minus CCT. All measurements were performed at baseline and during the follow-up visits. All surgeries were performed by a single surgeon (CET). The follow-up protocol and the surgical technique are described in detail elsewhere [12].

### 2.1. Statistical Analysis

The main outcome variable was ECD change at 1 year postoperatively. Descriptive statistics were performed to characterize the study sample. The mean and SD were calculated for the continuous variables. The Wilcoxon signed-rank test was used to compare mean values before and after cataract surgery. Percentage ECD change from baseline was considered as the dependent variable in the univariate and multivariate models. The average of nasal and temporal AODs and TISA measurements were used for regressions. The regression coefficients (*B*), coefficients of determination (*R*^2^), and statistical significance (*p* value) were reported. Two-tailed significance values were given with *p* < 0.05 regarded as significant. The statistical software package Stata 15.1 (StataCorp., College Station, TX) was used for all analyses.

## 3. Results

**Figure 1:**
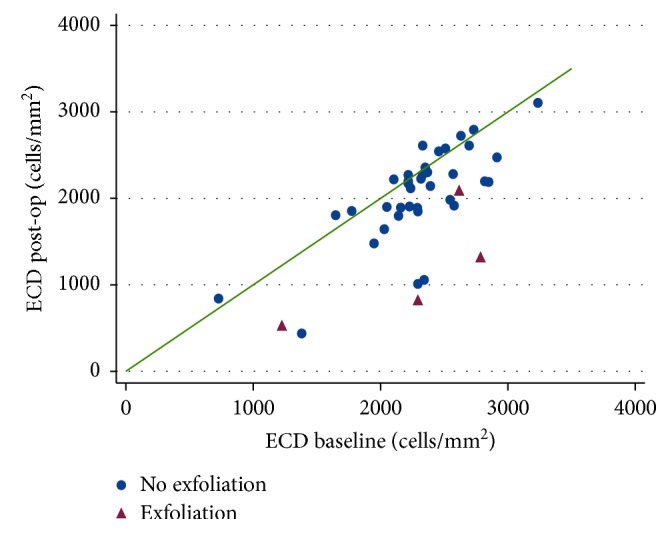
Pre-post graph of changes in endothelial cell density (ECD). The green line represents no effect.

Based on inclusion and exclusion criteria, we analyzed data from 44 patients. Patient demographics and baseline ocular characteristics are summarized in Table 1. The overall mean (SD) ECD was 2274 (463) cells/mm^2^ and decreased to 1964 (613) cells/mm^2^ one year after surgery (*p* < 0.001).  Table 2 summarizes dimensional changes of the AC. Coexistence of PAC or PACG and exfoliation syndrome (XS) were observed in 4 cases (9.1%). In the univariate analysis, both age and XS were significantly associated with the decrease in ECD (*p*=0.04 and *p* < 0.001, respectively). The baseline mean (SD) ECD was 2279 (445) and 2230 (700) cells/mm^2^, respectively, in patients with and without XS (*p*=0.84). The pre-post scatter graph (Figure 1) shows the individual changes in ECD for both groups. In the multivariate model, XS remained significantly associated with ECD changes, while age did not.  Table 3 reports the results of univariate and multivariate regressions.  Table 4 summarizes ECD change in angle closure and primary open-angle glaucoma after phacoemulsification. In both groups, a significant ECD loss was observed (*p* < 0.001).

## 4. Discussion

Our results show that PE in PAC and PACG is associated with a mean ECD reduction of 14%. We showed that the XS coexistence caused an additional change of −38% in ECD compared to patients without XS.

XS is an age-related systemic disease characterized by the accumulation of a fibrillar extracellular material over various tissues including ocular structures. XS is considered the most common identifiable cause of open-angle glaucoma even if the association of XS and angle closure is not rare [21, 22]. XS is also associated with a lower ECD compared to age-matched controls [23].

PE is a well-known cause of ECD reduction mainly due to the effect of the dissipated energy produced by instrument tip, the fluid shear stress, and intraocular manoeuvres. Walkow et al. found that, among preoperative and intraoperative parameters, the only factor associated with ECD decrease was short axial length [24].

In a Chinese population with occludable angles, Ko et al. showed that ECD decrease after phacoemulsification was negatively associated with axial length (the shorter the globe, the higher the cell loss) and with postoperative IOP within 24 hours [17].

This result agrees with the report of Hwang et al. who found that anterior chamber depth <2.5 mm, the distance between the anterior cornea vertex to the anterior lens vertex, and a denser cataract were associated with ECD decrease [25].

Clinically, short axial length and swallow anterior chamber are usually highly correlated. In our case series, patients had a mean baseline AD of 2.07 mm that, together with a CCT of 549 *μ*m, resulted in an ACD of 2.62 mm. This measurement is close to the high-risk cutoff value previously mentioned [25] However, in our population of Caucasian patients, axial length, CCT, and preoperative ACD were not associated with EDC change.

Wirbelauer et al. reported that PE induced similar endothelial cell modification both in patients with and without exfoliation at six months [23]. Demircan et al. showed that the percentage change in ECD was statistically significantly higher in the exfoliation group than that in the control group [26]. In another study by Kaljurand and Teesalu, exfoliation had a negative impact on endothelial cells only in interaction with the overall phaco impact [27].

The main limitation is that we did not collect intraoperative variables such as the cumulative dissipated energy or irrigation volume. Intraoperative variables however are generally correlated with baseline ocular characteristics such as the lens status that was considered in our model [28].

It has been estimated that up to 9.3% of eyes with exfoliation have an occludable angle [22]. Rarely, exfoliation may cause phacodonesis and lens subluxation, causing a secondary angle closure [29, 30]. In our study, secondary angle closures were excluded regardless of the mechanism. The association of angle closure and exfoliation, in absence of clinically evident phacodonesis or lens subluxation, was not considered an exclusion criterion since it is not rare in the clinical setting [22].

Ianchulev et al. reported a significant ECD loss (−10%) in patients submitted to phacoemulsification for the treatment of cataract in primary open-angle glaucoma (Table 4) [20]. Additionally, they reported that age was associated with ECD decrease. In our study on PAC and PACG, a slightly higher ECD loss was observed. Remarkably, in our study, 25 eyes (58.1%) had clear lens, whereas in the study by Ianchulev et al., all the patients had visually significant cataracts that probably required higher PE energy. Although PE in angle closure could be technically challenging, in our study, a single senior surgeon (CET) with extensive experience in complex cataract extraction and glaucoma subspecialty training performed all the procedures. Therefore, the results may not be applicable in other settings in which procedures are performed by different surgeons [12].

Our findings show that PE for the treatment of PAC and PACG is associated with a significant reduction in ECD, and the XS presence at the preoperative visit was the strongest predictor of endothelial damage at one year. Therefore, a careful endothelial preoperative evaluation should be performed in this group of patients.

## Figures and Tables

**Table 1:** Baseline demographics and ocular characteristics.

Demographics	

Women	31 (70.5%)
Age (years)	71.6 (10.2)

Ocular characteristics	

Study eye was the right eye	23 (52.3%)
Glaucomatous optic neuropathy	22 (51.2%)
Exfoliation syndrome	4 (9.1%)
Clear lens	25 (58.1%)
Prior iridotomy	17 (38.6%)
IOP (mmHg)	20.4 (9.0)
BCVA (ETDRS letters)	69 (14)
Lens thickness (mm)	4.9 (0.3)
Central corneal thickness (*μ*m)	549 (34)
ECD (cells/mm^2^)	2274 (463)
Axial length (mm)	22.27 (0.98)

Data are number (%) or mean (standard deviation). IOP = intraocular pressure; BCVA = best-corrected visual acuity; ECD = endothelial cell density.

**Table 2:** Changes in anterior chamber ocular parameters.

	Baseline	Post-op	*p*
Aqueous depth (mm)	2.07 (0.29)	3.54 (0.38)	<0.001
Central corneal thickness (*μ*m)	553 (36)	555 (43)	0.399
Nasal SL-AOD (*μ*m)	189 (148)	534 (220)	<0.001
Temporal SL-AOD (*μ*m)	214 (155)	537 (254)	<0.001
Nasal SL-TISA (mm^2^)	0.065 (0.054)	0.166 (0.089)	<0.001
Temporal SL-TISA (mm^2^)	0.076 (0.063)	0.164 (0.099)	<0.001

SL = Schwalbe's line; AOD = angle-opening distance; TISA = trabecular iris space area. Statistical significance was analyzed by the Wilcoxon signed-rank test.

**Table 3:** Analysis of the association between demographic and anatomical parameters and endothelial cell density percentage change.

	Univariate			Multivariate		
	*B*	95% CI	*p*	*B*	95% CI	*p*
Age (years)	−0.65	−1.26 to −0.037	0.038	−0.17	−0.83 to 0.50	0.611
Male	−2.84	−17.07 to 11.40	0.690			
Axial length (mm)	10.63	−32.91 to 54.17	0.625			
Aqueous depth (mm)	−2.26	−25.16 to 20.65	0.843			
Pseudoexfoliation	−38.82	−57.96 to −19.67	<0.001	−36.00	−56.57 to −15.42	0.001
SL-TISA (mm^2^)	−71.42	−193.21 to 50.36	0.243			
SL-AOD (*μ*m)	−0.023	−0.07 to 0.02	0.335			
Lens thickness (mm)	13.43	−6.43 to 33.31	0.179			
Clear lens	9.43	−3.69 to 22.54	0.154	6.63	−6.38 to 19.65	0.309
Pre-op IOP (mmHg)	0.012	−0.73 to 0.76	0.973			
Antiglaucoma drugs (*n*)	2.88	−1.55 to 7.30	0.197			

Statistical significance was analyzed by the multivariate linear regression model: *p*=0.0027; *R*^2^ = 0.33.

**Table 4:** Comparison of endothelial cell density change in primary angle closure and in open-angle glaucoma.

	Primary angle closure			Open-angle glaucoma		
	Preoperative	12 months	Difference	Preoperative	12 months	Difference
ECD (cells/mm^2^)	2274 (463)	1964 (613)	−310	2453 (359)	2186 (479)	−267
*p* value	<0.001			<0.001		

Data are mean (SD). ECD = endothelial cell density. Data for open-angle glaucoma were obtained from the work of Ianchulev et al. [20].

## Data Availability

The data used to support the findings of this study are available from the corresponding author upon request.
